# Drug Target Prediction Based on the Herbs Components: The Study on the Multitargets Pharmacological Mechanism of Qishenkeli Acting on the Coronary Heart Disease

**DOI:** 10.1155/2012/698531

**Published:** 2012-03-08

**Authors:** Yong Wang, Zhongyang Liu, Chun Li, Dong Li, Yulin Ouyang, Junda Yu, Shuzhen Guo, Fuchu He, Wei Wang

**Affiliations:** ^1^Beijing University of Chinese Medicine, Bei San Huan Dong Lu 11, ChaoYang District, Beijing, China; ^2^State Key Laboratory of Proteomics, Beijing Proteome Research Center, Institute of Radiation Medicine, Beijing 100850, China

## Abstract

In this paper, we present a case study of Qishenkeli (QSKL) to research TCM's underlying molecular mechanism, based on drug target prediction and analyses of TCM chemical components and following experimental validation. First, after determining the compositive compounds of QSKL, we use drugCIPHER-CS to predict their potential drug targets. These potential targets are significantly enriched with known cardiovascular disease-related drug targets. Then we find these potential drug targets are significantly enriched in the biological processes of neuroactive ligand-receptor interaction, aminoacyl-tRNA biosynthesis, calcium signaling pathway, glycine, serine and threonine metabolism, and renin-angiotensin system (RAAS), and so on. Then, animal model of coronary heart disease (CHD) induced by left anterior descending coronary artery ligation is applied to validate predicted pathway. RAAS pathway is selected as an example, and the results show that QSKL has effect on both rennin and angiotensin II receptor (AT1R), which eventually down regulates the angiotensin II (AngII). Bioinformatics combing with experiment verification can provide a credible and objective method to understand the complicated multitargets mechanism for Chinese herbal formula.

## 1. Introduction

Coronary heart disease (CHD) remains the single leading cause of death for adults worldwide [1]. Effective prevention and therapy for CHD poses a major challenge to the entire medical community. There exists a strong demand to continue searching for both safe and efficacious products to combat this emerging health epidemic. Traditional Chinese medicine (TCM) has fought against CHD and its related diseases for more than 1000 years and has accumulated thousands of herbal formula as well as clinical literatures, it has been considered to have huge potential as an information source and starting point for the development of CHD products [2]. Meanwhile, more and more patients all over the world take TCM as a complementary and alternative avenue to treat CHD.

However, how herbal formula work and what are their drug targets are still unclear by now. Many studies have focused on active monomer of herbs to explain their therapy mechanism [3], but apparently there are significantly different characteristics between active monomer and herbal formula as whole. Active monomer may have a clear target, such as receptors, enzymes, ion channels, transmembrane signal transduction molecules, mostly acting on single-target, but Chinese herbal formula composed of diverse, complex components, its comprehensive pharmacological effects is accumulated by many active monomers through multichannel and multitargets [4]. How to determine the multitargets from such a complex biological process is a challenge to TCM.

Coronary heart disease (CHD) is now a heavy burden on the society and families in both industrialized and developing countries, and some herbal formula present a definitely clinical effect on it, so it presents a better example and context for investigating the efficacy and the drug targets in TCM.

The ancient TCM Qishenkeli (QSKL), prepared from a basic formula of six Chinese herbs (Radix Astragali Mongolici, salvia miltiorrhiza bunge, Flos Lonicerae, Scrophularia, Radix Aconiti Lateralis Preparata, and Radix Glycyrrhizae, etc.) is widely produced in China in accordance with the China Pharmacopoeia standard of quality control [5] and is commonly used in routine treatment of CHD of clinical practice in China. It contains large-scale epidemiological survey in the randomized controlled clinical trials proved that it has a definite effect on improving heart function [6], while a lot of studies are carried out to investigated in active monomers among them and made great progress, for example, Astragalus Polysaccharide (APS, monomer of Radix Astragali Mongolici) is found has effect on cardiac chymase activities [7], tanshinone IIA (monomer of salvia miltiorrhiza bunge) is found in cardioprotective effects and attenuating myocardial hypertrophy [3], but as mentioned before, monomer pharmacological effects cannot present overall efficacy of the whole formula, studies involved all the compounds are rarely carried out.

In recent years, people develop some bioinformatic methods to infer drug target interactions [8–13]. These methods provide opportunities to reveal the underlying molecular mechanism of TCM. Recent advances on the databases cataloging chemical components of herbs and the interactions between drugs and targets enhance the feasibility of predicting the herbs drug targets.

DrugCIPHER-CS is an efficient drug target prediction method which is recently presented by Zhao and Li [14], and in this paper, we use it to predict the potential targets of QSKL's compositive compounds. This method is based on the principle that (i) drugs with similar chemical structure tend to bind functionally related proteins and (ii) functional relationship between the proteins can be measured by their distance in the protein interaction network. For a query drug, each protein in the protein interaction network will be assigned a score by DrugCIPHER-CS which describes the importance of the protein to the activity of the drug, and proteins with high scores will be hypothesized as this query drug's potential targets.

This paper presents an idea that multi targets for herbs should be investigated by combing bioinformatics and experimental verification to finally determine drug targets. Firstly, herbal components are investigated by data mining from database; secondly, bioinformatics is applied to predict the drug target for all compounds based the principle of that similar structural has similar function, then bioinformatics including GO function analysis are used to look for the pathway that the proteins belong. Finally, experimental verification is taken to confirm how and what the herbs work on the body, thus to provide a credible method to investigate the complicated multitargets mechanism for herbs.

## 2. Methods

### 2.1. Drug Targets Prediction

In this paper, we use drugCIPHER-CS to predict drug targets of QSKL's compositive compounds. DrugCIPHER-CS recently presented by Zhao and Li [14] achieves good prediction performance and can infer drug targets in the genome wide scale. This method is based on the hypotheses that (i) drugs with similar chemical structure usually bind functionally related proteins and (ii) functional relationship between the proteins can be measured by their distance in the protein interaction network. Given a set of known drug- (drug-space) target (target-space) interactions, for a query drug and a candidate target gene, drugCIPHER-CS will measure the likelihood of their interaction based on the correlation between the query drug's structure similarity vector with the drug space and the candidate gene's functional similarity vector with the target space. For a query compound, drugCIPHER-CS will prioritize the proteins in the protein interaction network (i.e., candidate proteins) according to the order of the decreasing drug target interaction likelihood, and the candidate proteins with high likelihood will be hypothesized as the potential drug targets (Please refer to paper [14] for more details of DrugCIPHER-CS).

Here, known drug target interactions are obtained from DrugBank database (version: May, 2011) [15]. We only use those drug-target interactions whose drugs are FDA-approved and have InChI identifiers [16] and whose targets are human genes/proteins. In total, we obtain 4299 interactions between 1109 drugs and 1138 targets. The chemical structure similarity is calculated based on compounds' MOLPRINT 2D descriptors and Tanimoto coefficient [17]. The human protein interaction network is constructed by integrating the protein interaction data from HPRD (release 9.0) [18], BioGRID (version: 3.0.66) [19], IntAct (version: 20100628) [20], MINT (version: 20100505) [21], DIP (version: 20100614) [22], and PDB provided by Gibson and Goldberg [23]. In total, there are 102131 interactions between 11654 proteins in the protein interaction network.

### 2.2. Degree and betweenness Centrality in the Protein Interaction Network

A protein's degree is defined as the number of its direct interaction partners in the protein interaction network. The betweenness centrality of protein *n* is computed as

(1) $$\begin{array}{cc} B\left(n\right) & =\frac{\left(\sum_{s\neq n\neq t}(\sigma_{st}\left(n\right)/\sigma_{st})\right)}{\left(\left(N-1\right)\left(N-2\right)/2\right)}, \end{array}$$

where *σ*_*st*_ denotes the number of the shortest paths between protein *s* and protein *t* in the protein interaction network, *σ*_*st*_(*n*) denotes the number of the shortest paths across protein *n* between protein *s* and protein *t*, and *N* is the total number of proteins in the protein interaction network.

Both degree and betweenness centrality can measure a protein's topological importance in the network. The larger a protein's degree/betweenness centrality is, the more important the protein is in the protein interaction network.

### 2.3. CHD Model Preparation

CHD is induced by direct coronary ligation as described before [24]. Briefly, Sprague-Dawley (SD) rats are anaesthetized with pentobarbital sodium (1%, 50 mg kg^−1^ intraperitoneally). The trachea of each rats is intubated per orally with a plastic tube connected to a respirator (Kent Scientific 325, China) set at a stroke volume of 3 mL kg^−1^, respiratory ratio: 2 : 1, and a rate of 80 strokes min^−1^. After left thoracotomy and exposure of the heart, the left anterior descending coronary artery (LAD) is ligated with a 5–0 polypropylene suture (Surgipro, CT, USA) directly proximal to its main branching point. Control groups are made following an identical procedure but without the actual tying of the polypropylene suture. Thereafter, the thorax is closed and as soon as spontaneous respiration is sufficient, the rats are extubated and are allowed to recover under a heated lamp. They are fed a standard diet and water and are maintained on a 12-hour Light-and-dark cycle. After ECG testing, rats that averaged QT-interval prolongation in three precordial leads are included in the study. The QSKL group is treated for 28 days by daily oral gavage with total daily dosages of 508 mg/kg of the concentrated QSKL (Beijing university of Chinese Medicine, Beijing, China) dissolved in water. The control and model groups receive the same volume water via oral gavage as the QSKL vehicle. At the end of the study, all animals are anaesthetized using pentobarbital sodium following an overnight fast. Blood samples are collected via abdominal aorta puncture, place on ice, and allow to clot. After centrifugation, serum is collected, aliquoted, and stored at −80°C until analysis of each indicator within a short period of time.

### 2.4. Echocardiographic Assessment of LV Function

Echocardiography is used to detect Left ventricular end-systolic diameter (LVEDs), Left ventricular end-diastolic diameter (LVEDd), ejection fraction (EF), fractional shortening (FS), and other indicators. A PST 65A sector scanner (8-MHz probe) is used, which generates two-dimensional images at a frame rate ranging from 300 to 500 frames/s. LV dimension (LVD) is measured by M model, and fractional shortening (FS%) is calculated by the following equation:

(2) $$\text{FS\%}\;=\;\left[\frac{\text{LVEDd}-\text{LVEDs}}{\text{LVEDd}}\right]*100\text{\%}.$$

### 2.5. Preparation and Dose Consideration of Concentrated QSKL

The QSKL used in this study is manufactured by Beijing university of Chinese medicine (Beijing, China) using the six Chinese herbs at a composition of 460 g Radix Astragali Mongolici, 230 g salvia miltiorrhiza bunge, 160 g Flos Lonicerae, 160 g scrophularia, 140 g Radix Aconiti Lateralis Preparata, and 90 g Radix Glycyrrhizae. Briefly, the residue of Radix Astragali Mongolici is mixed with all salvia miltiorrhiza bunge, Flos Lonicerae, scrophularia, and Radix Glycyrrhizae, follow by extraction with hot water (twice, 2 hr each). The water extract is then concentrated to form a paste, and the ethanol is added for 24 hr, the filtration is collected to form the final product. Based on the recommended daily human dosage of 20 g/d, according to the equivalent conversion between animal and people by body surface area, dosage of 508 mg/kg is chosen in present study.

### 2.6. Biological Parameter Detection

#### 2.6.1. Measurement of Serum Indicators by Elisa

Levels of serum indicators (appeared in predicting target) are quantified in duplicate using commercial ELISA kits (Abcam Inc., Cambridge, MA, USA). Each assay is performed following the kit instructions. Standards at a series of concentrations are run in parallel with the samples. The concentrations in the samples are calculated in reference to the corresponding standard curves and expressed as ng/mL.

#### 2.6.2. Measurement of Indicators by Western Blot

The serum are homogenised in RIPA buffer (50 mM TrisHCl pH7.4, 150 mM NaCl, 2 mM EDTA, 1% NP-40, 0.1% SDS) and total protein is extracted from this homogenate. The protein concentration in each sample extract is measured using a protein assay kit (Pierce; Rockford, IL, USA) and then is adjusted to the same value in all samples with 2X 4% SDS sample buffer. The samples are boiled for 5 min followed by loading on a 7.5% SDS-PAGE gel (30 mg protein/10 mL per well) for electrophoresis using a Bio-Rad mini gel apparatus at 100 V for 2 hours. The fractionated protein on the gel is transferred onto a NC membrane (Millipore) and electrophoresed at 300 mA for 90 min. The membrane is first probed with AT1R primary antibody (antiangiotensin II type 1 receptor antibody, ab18801, Abcam, 1 : 500) and secondary antibody (donkey polyclonal secondary antibody to rabbit IgG-HRP, ab97064, Abcam, 1 : 5000), and then treated with ECL (ECL Plus western blotting detection reagent, GE Healthcare) for 1 min at room temperature. The bands in the membrane are visualized and analyzed using UVP BioImaging Systems. After obtaining the AT1R blot density, the membrane is then treated using restore western blot stripping buffer (Thermo Scientific) to remove the AT1R signal, followed by probing with glyceraldehyde-3-phosphate dehydrogenase (GAPDH) primary antibodies (GAPDH mouse monoclonal IgG, ab8245, Abcam, 1 : 2000) using the same process as the AT1R antibody to get the AT1R and GAPDH blot densities. The final reported data are the normalized AT1R band densities by GAPDH.

#### 2.6.3. Measurement of Indicators by Immunohistochemistry (IHC)

An avidin-biotin-peroxidase complex commercial method (R&D) is used for immunohistochemistry. Briefly, 4-mm-thick paraffin wax sections are mounted on slides, which are dried for 30 minutes in an oven (60–70°C) and deparaffinized in xylene. The slides are then placed in changes of ethanol for 2 minutes each. Washing in buffer solution is performed between steps. The slides are then placed in 3% hydrogen peroxide for 15 minutes. And then are subsequently incubated in avidin block for 15 minutes, biotin block for 15 minutes, primary antibody (Ang II antibody, Phoenix Pharmaceuticals Inc. or Anti angiotensin II type 1 receptor antibody, ab18801, Abcam) for 12 hours at 4°C, and biotinylated secondary antibody for 1 hours. The reagent incubation is performed with streptavidin peroxidase for 15 minutes. A 1-minute Mayer's hematoxylin counterstain is used. The slides are dehydrated, cleared with xylene, and mounted with permanent mounting medium. Finally, integral optical density (IOD) of pictures is analyzed by IPP6.0 software.

### 2.7. Statistical Analysis

Data analyses are performed by one-way ANOVA using SAS 9.2 statistical software (SAS Institute, NC, USA). *P* < 0.05 was considered statistically significant. Results are presented as mean values with their standard deviation.

## 3. Results

### 3.1. Drug Target Prediction and Analyses

In order to reveal the underlying molecular mechanism of QSKL, we firstly use bioinformatic method to infer the targets of its chemical components.

**Figure 1:**
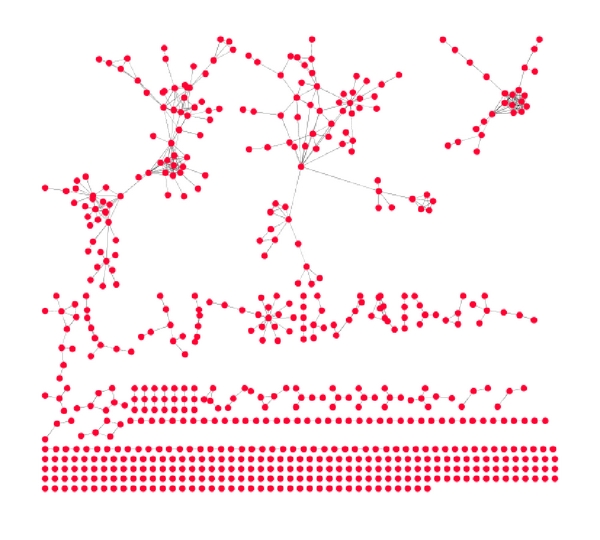
The protein interaction network consists of top 0.1% candidate target genes.

By use of literature curation, we determine QSKL's 231 compositive compounds. Then we use drugCIPHER-CS method [14] to infer their potential targets (Supplementary Table 1 avaliable online at doi: 10.1155/2012/698531). drugCIPHER-CS published recently by Zhao and Li achieves good performance for predicting the targets of drugs and can infer targets in the genome-wide scale [14]. For each compositive compound, drugCIPHER-CS prioritizes its candidate targets according to the order of the decreasing possibility being targeted by the compound. When we choose top 1% candidate targets, we obtain 3725 candidate target genes for 207 compositive compounds which have clear chemical structures. Average, one target gene is shared by 6.5 compounds. When we choose top 0.1% predicted targets, we obtain 639 target genes. Average, one gene is targeted by 3.6 compounds. As shown in Figure 1, there are 510 protein interactions between these 639 top 0.1% candidate targets (Figure 1).

By comparing with the known cardiovascular disease-related drug targets (i.e., the known targets of drugs whose ACT code uses “C” as the first level) in DrugBank [15], we find both top 0.1% and top 1% candidate targets are significantly enriched with known cardiovascular disease-related targets (upper-tailed *P* value of hypergeometric cumulative distribution is 2.03*E* − 10 for top 0.1% and 2.05*E* − 08 for top 1% candidate targets). And the corresponding enrichment extent of top 0.1% candidate targets is higher than that of top 1% targets.

After obtaining the potential targets for the QSKL's chemical components, we analyze the enriched KEGG biological pathways [25] (version: 2009.11) among these potential targets. In total we find 16 significantly enriched pathways among top 0.1% candidate targets (Table 1), including the pathways of neuroactive ligand-receptor interaction, aminoacyl-tRNA biosynthesis, calcium signaling pathway, glycine, serine and threonine metabolism, Renin-angiotensin system, and so on. The importance of Neuroactive ligand-receptor interaction in the development and progress of cardiovascular disease processes such as CHD is well known, The key protein in this pathway such as Adrenergic receptor, Angiotensin receptor, Calcitonin receptor-like, Neurotensin receptor are closely related to the cardiac function. The pathway of Aminoacyl-tRNA biosynthesis plays a important roles in cardiovascular angiogenesis [26], The relationship between calcium signaling pathway and CHD is confirmed, and calcium antagonists have been widely used in clinical to inhibit extracellular calcium influx, reducing the concentration of intracellular calcium and lower myocardial contractility [27]. Glycine, serine, and threonine metabolism mainly provide the ATP for myocardial contractility [28]. Renin-angiotensin system plays a central role in the deterioration of cardiovascular function [29].

Also, we research the functional distribution of these candidate targets (Table 2). The significantly enriched gene ontology (GO) functional annotations [30] (version: 20111103) of these targets include cellular amino acid metabolic process, biosynthetic process, small molecule metabolic process, cellular nitrogen compound metabolic process and circulatory system process, indicating the QSKL intervening in these pathological progresses. These enriched pathways and GO functional annotations provide important clues for understanding the molecular mechanism of QSKL.

In addition, by checking the degree and betweenness centrality of these candidate target genes in the protein interaction network, we find these candidate targets are significantly depleted with the proteins with the highest degree or betweenness centrality (Table 3). And the depletion extent for top 0.1% candidate targets is larger than that for top 1% candidate targets. That is, these QSKL's candidate target genes do not tend to be topologically the most important in the protein interaction network. This result is consistent with Hase et al.'s conclusion that known human drug targets tend to be less connected nodes in the network [31]. The TCM with multiple chemical components targets multiple less-connected nodes, which may produce greater synergetic efficacy and fewer side effects.

### 3.2. Experimental Validation

#### 3.2.1. Model Evaluation

**Figure 2:**
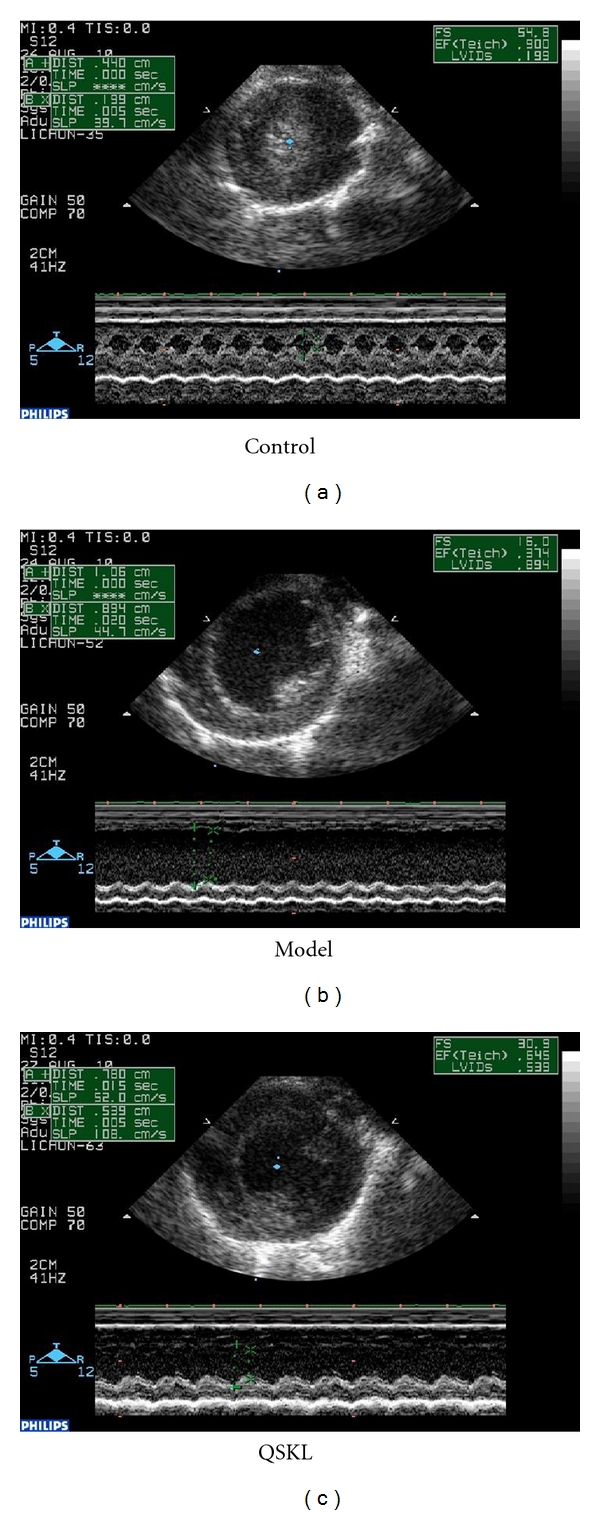
The cardiac function in different groups. Control groups showed a high EF value, while abnormal ventricular wall movement in model group is seen, in QSKL group, EF value recovers in some extent.

28 days after surgery, echocardiography showed that EF and FS values in the model group were significantly different (*P* < 0.05). EF value of ligation rats in model group dropped down to 49.03% compared with control group, suggesting a steady CHD model is established. After treated by QSKL for 28 days, the EF value recovers by 37.62% compared with model group (Figure 2).

#### 3.2.2. Predicting Pathway Validation

The importance of neurohormonal activation in the development and progress of cardiovascular disease processes such as CHD is well known, and the renin-angiotensin system plays a central role in this [32].The chronically activated renin-angiotensin aldosterone system (RAAS) is believed to contribute significantly to the deterioration of cardiovascular function, Inhibitors of it have been routinely used to treat patients with CHD [29]. In this paper, RAAS are selected as example and context to validate predicting pathway. Critical indicators in RAAS pathway are detected to test the accuracy of the predicting pathway, we carry out series experiments to validate them including Elisa, IHC, and westernrblot.

**Figure 3:**
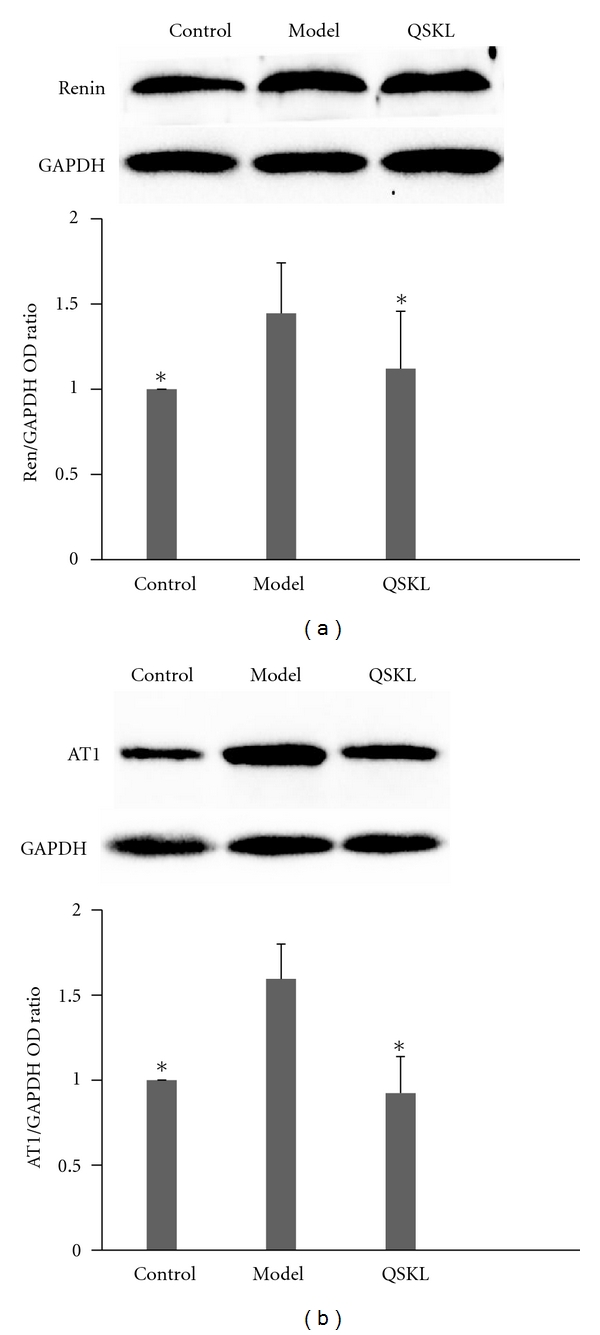
QSKL significantly lowers rennin and AT1R in CHD rats. (a) Rennin levels in different group; (b) AT1 levels in different group. Data are analyzed by one-way ANOVA. *P* < 0.05 indicates statistical significance. Results are presented as mean values with their standard deviation (*n* = 20). *Differed significantly from model (*P* < 0.05).

The western blot of renin shows that at the end of the study, the serum renin in model group increases by 45% (*P* < 0.05) compared with control, after treated by QSKL for 28 days, the level of renin shows a 22.76% reduction compared with model group (*P* < 0.05), which had no statistical significance when compared to the control (Figure 3(a)).

**Figure 4:**
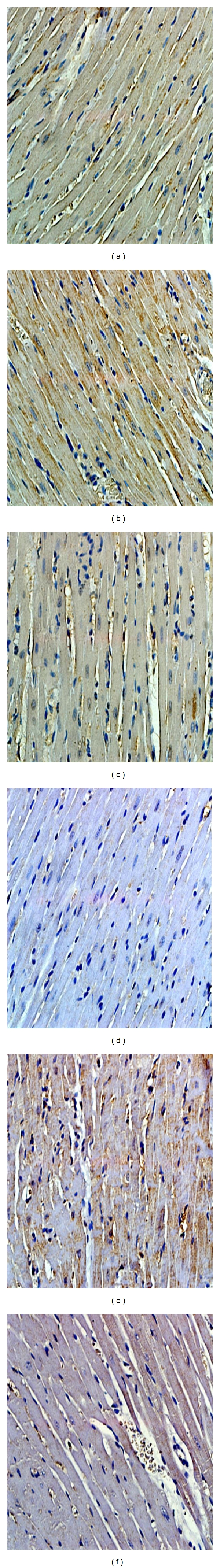
IHC results of Ang II and AT1R in control, model and QSKL group. (a) Cardiac Ang II expression in control group. (b) Upregulating cardiac Ang II expression in model group. (c) QSKL can reduce the level of the Ang II. (d) Cardiac AT1R expression in control group. (e) Disorders in myocardial cells, upregulating cardiac AT1 expression in model group. (f) QSKL can decrease the level of the AT1.

**Figure 5:**
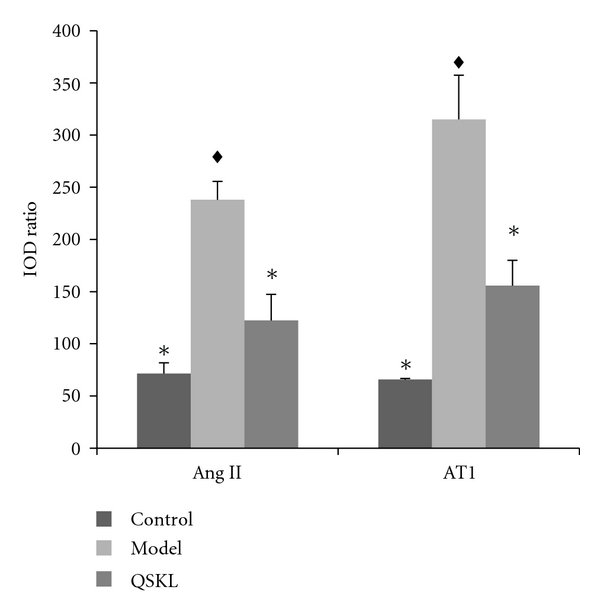
Semiquantitative determination of Ang II and AT1R expression with IHC in different groups. ^*∗*^Differed significantly from model (*P* < 0.05).

Both Elisa and IHC results show that the levels of Ang II in model group upregulated by 27.88% compared with control (*P* < 0.05), after treated by QSKL for 28 days, a 16.59% reduction are detected in QSKL group compared with model (*P* < 0.05),which almost return to the level of the control (Figures 4 and 5, Table 4).

AT1R is thought to be a better target to cure the CHD. The AT1R in model group up regulated by 59.00% compared with control. In QSKL group, its level decreases by 42.12% compared with model, which has no significant difference with control (Figures 3(b), 4, and 5). The level of serum aldosterone (ALD) in each group does not show any significant difference.

## 4. Discussion

At present, monomer in herbs is usually applied to explain the pharmacological efficacy of a whole Chinese herbal formulation. In fact, it did not present the multitarget characteristic of the multi component Chinese herbal formulation. If the multi targets can be predicted according to chemical structure of its composition through the bioinformatics, and experiments to verify the results, things will be go easy and concise to confirm herbs pharmacological mechanisms.

With the development of high-throughput drug screening and structural analysis technology, the chemical compositions of formulation are gradually revealed, mature database of the chemical composition of Chinese herbs are gradually established, and the identification of the chemical structure makes it possible to predict drug targets by investigating the relations between the drug and the biomarkers proteins. As the development of system biology, bio formations technique becomes more and more mature. Its advantages are very applicable to the complex correlativity study of compound in herbs and the drug targets.

In this paper, we take drugCIPHER-CS to predict the target of QSKL which has been used for treating CHD effectively for thousand years. Five pathways were predicted as a main way that the QSKL may act on. RAAS was selected to elaborate the pharmacological mechanism of QSKL. After experimental verification, more than one target was verified including renin, Ang II, AT1, which can elaborate the characteristic of the milt-target of Chinese herbal formulation.

The chronically activated renin-angiotensin-aldosterone system (RAAS) is believed to contribute significantly to the deterioration of cardiovascular function. In the pathway, angiotensin II has critical roles including the regulation of blood pressure, vasoconstriction, increasing aldosterone secretion, amplifying sympathetic activity, increasing sodium retention, as well as lots of other actions. It is considered a factor in virtually every form of CHD, and it is applied as a therapeutic target in hypertension and chronic heart failure. Numerous researches focus on its inhibitors to provide clinical drug for CHD. Among them, Antagonists to AT1R and angiotensin-converting enzyme inhibitors (ACEI) have been routinely used to treat patients with CHD [33, 34]. Both experimental and clinical studies have shown that ACEI, besides inhibiting the concentration of Ang II, could have desirable effects by down regulating the bradykinin [35], moreover, patients levels of angiotensin II have a tendency to return to pre treatment levels after long-term ACEI treatment [36]. Since ACEI do not seem to have complete protective effects against the detrimental effects of Ang II, AT1-receptor blockers may offer advantages relative to ACEI by effectively blocking the AT1-receptor, which mediates all known detrimental effects of Ang II. The AT1R mediates the majority of classical biological functions of Ang II [37] and plays a critical role in the control of regulation of blood pressure, vasoconstriction, increasing aldosterone secretion, amplifying sympathetic activity, and so forth. All the AT1-receptor antagonists in routinely clinical use are extremely well tolerated. Since AT1R blockers for the treatment of cardiovascular disease seem very promising, indeed, the AT1R has been regarded as an important target for cardiovascular treatment. In our research, the QSKL can significantly down regulated the level both Ang II and AT1R, indicating a same efficacy as AT1 agonists. Besides, the QSKL can lower the RAAS activation form the very beginning-the renin. Renin is an aspartyl-protease enzyme produced and activated within the juxtaglomerular (JG) cells of the afferent arteriole in the kidney. Through Angiotensin I, it can activate Ang II which is the primary biologically active hormone of the renin-angiotensin system as referring before. Renin secretion is the critical rate-limiting step in the activity of the entire system [38]. Because of this, QSKL regulating renin secretion are of particular interest and importance in understanding its collaboration effect with Ang II as well as understanding therapeutic targets for CHD. ALD seems not to change, which is consistent with the published papers [39]. “ALD breakthrough” is thought to be its important mechanism.

 To sum up, this paper presents an idea that the study of multi target for Chinese herbal formula are carried out based on the known chemical composition of herbs both by bioinformatics and experimental verification. We take the research of QSKL effect on CHD as an example. And the results show it can act on CHD in multi targets, especially in renin and AT1, eventually decrease the level of the Ang II, which can treat CHD efficiently. From this, a credible and objective method can be provided to understand and confirm the complicated multi targets mechanism for Chinese herbal formulation.

But some problems still exist. For example, in predicting drug targets, the distribution and metabolism of herbal formulation in the body are not taken into consideration in our research; we presume all components of herbal formulation compounds are absorbed and utilized; improvement should be made in our future work.

## Figures and Tables

**Table 1:** Significantly enriched KEGG biological pathways among top 0.1% candidate target genes of QSKL compositive compounds.

KEGG pathway number and name	*P* value^a^	Coverage^b^
hsa04080 neuroactive ligand-receptor interaction	1.17*E* − 10	0.1358
hsa00970 aminoacyl-tRNA biosynthesis	1.54*E* − 08	0.3171
hsa04020 calcium signaling pathway	1.34*E* − 06	0.1348
hsa00260 glycine, serine, and threonine metabolism	3.90*E* − 04	0.2258
hsa04614 renin-angiotensin system	7.51*E* − 04	0.2941
hsa00290 valine, leucine, and isoleucine biosynthesis	1.09*E* − 03	0.3636
hsa00590 arachidonic acid metabolism	1.12*E* − 03	0.1552
hsa00350 tyrosine metabolism	4.42*E* − 03	0.1522
hsa04260 cardiac muscle contraction	1.02*E* − 02	0.1125
hsa00330 arginine and proline metabolism	1.07*E* − 02	0.1296
hsa04270 vascular smooth muscle contraction	1.13*E* − 02	0.0960
hsa00250 alanine, aspartate, and glutamate metabolism	1.22*E* − 02	0.1613
hsa04144 endocytosis	2.32*E* − 02	0.0802
hsa04115 p53 signaling pathway	3.69*E* − 02	0.1014
hsa00071 fatty acid metabolism	4.09*E* − 02	0.1190
hsa00591 linoleic acid metabolism	4.11*E* − 02	0.1379

^a^A pathway is significantly enriched with candidate target genes when its corresponding upper-tailed *P* value of hypergeometric cumulative distribution is smaller than 0.05. The pathways are ranked according to the order of the increasing *P* values. ^b^The coverage for each pathway is referred to as the fraction of candidate target genes among all the pathway member genes.

**Table 2:** Significantly enriched GO term among top 0.1% candidate target genes of QSKL compositive compounds.

GO term ID	GO term name	*P* value^a^
GO:0006520	Cellular amino acid metabolic process	1.99*E* − 13
GO:0009058	Biosynthetic process	4.32*E* − 09
GO:0044281	Small molecule metabolic process	2.55*E* − 08
GO:0034641	Cellular nitrogen compound metabolic process	2.27*E* − 07
GO:0003013	Circulatory system process	1.40*E* − 06
GO:0006399	tRNA metabolic process	6.43*E* − 06
GO:0007267	Cell-cell signaling	1.60*E* − 04
GO:0006950	Response to stress	1.88*E* − 04
GO:0006412	Translation	3.70*E* − 04
GO:0042592	Homeostatic process	4.40*E* − 04
GO:0055085	Transmembrane transport	4.90*E* − 04
GO:0071941	Nitrogen cycle metabolic process	7.37*E* − 04
GO:0007568	Aging	7.90*E* − 04
GO:0006810	Transport	9.10*E* − 04
GO:0050877	Neurological system process	2.35*E* − 03
GO:0006461	Protein complex assembly	1.75*E* − 02
GO:0019748	Secondary metabolic process	1.99*E* − 02
GO:0065003	Macromolecular complex assembly	4.30*E* − 02

^a^The top 0.1% candidate target genes are significantly enriched with genes annotated with a GO term when its corresponding upper-tailed *P* value of hypergeometric cumulative distribution is smaller than 0.05. These GO terms are ranked according to the order of the increasing *P* values.

**Table 3:** The depletion analyses of proteins with the highest degree/betweenness centrality among top 0.1% and top 1% candidate target-genes.

	Lower-tailed *P* values of hypergeometric cumulative distribution	
	Top 0.1% candidate targets	Top 1% candidate targets

The proteins with the highest degree^a^	4.97*E* − 07	3.89*E* − 06
The proteins with the highest betweenness centrality^a^	4.97*E* − 07	3.24*E* − 05

^a^The proteins with the highest degree/betweenness centrality are referred to as those with top 5% degree/betweenness centrality in the protein interaction network.

**Table 4:** The change in indicators related to renin-angiotensin-aldosterone system (mean values with their standard deviation, *n* = 20).

	Control		Model		QSKL	

	Mean	SD	Mean	SD	Mean	SD
Ang II (×10^−6^ *μ*g/mL)	165.59*	21.352	211.28	19.853	176.71*	17.661
ALD (×10^−3^ *μ*g/mL)	208.85	47.953	220.32	20.608	236.49	32.965

QSKL: Qishenkeli; *mean values are significantly different from model (*P < 0.05).

## References

[1] He J, Gu D, Wu X (2005). Major causes of death among men and women in China. *New England Journal of Medicine*.

[2] Ferreira AS, Lopes AJ (2011). Chinese medicine pattern differentiation and its implications for clinical practice. *Chinese Journal of Integrative Medicine*.

[3] Tu EY, Zhou YG, Wang ZH, Liang QS, Yang GT (2009). Effects of tanshinone II A on the myocardial hypertrophy signal transduction system protein kinase B in rats. *Chinese Journal of Integrative Medicine*.

[4] Xu H, Chen KJ (2010). Herb-drug interaction: an emerging issue of integrative medicine. *Chinese Journal of Integrative Medicine*.

[5] Hong C, Wang Y, Lou J, Liu Q, Qu H, Cheng Y (2009). Analysis of myocardial proteomic alteration after Qishenyiqi formula treatment in acute infarcted rat hearts. *China Journal of Chinese Materia Medica*.

[6] Dai GH, Zhang BL, Guo ZX (2007). Application of central randomized system in project of clinical trial for secondary prevention of myocardial infarction by Qishen Yiqi Drop Pill. *Chinese Journal of Integrated Traditional and Western Medicine*.

[7] Chen W, Yu MH, Li YM, Chen WJ, Xia YP (2009). Beneficial effects of astragalus polysaccharides treatment on cardiac chymase activities and cardiomyopathy in diabetic hamsters. *Acta Diabetologica*.

[8] Parsons AB, Brost RL, Ding H (2004). Integration of chemical-genetic and genetic interaction data links bioactive compounds to cellular target pathways. *Nature Biotechnology*.

[9] Lamb J, Crawford ED, Peck D (2006). The connectivity map: using gene-expression signatures to connect small molecules, genes, and disease. *Science*.

[10] Campillos M, Kuhn M, Gavin AC, Jensen LJ, Bork P (2008). Drug target identification using side-effect similarity. *Science*.

[11] Nidhi, Glick M, Davies JW, Jenkins JL (2006). Prediction of biological targetsfor compounds using multiple-category bayesian models trained on chemogenomics databases. *Journal of Chemical Information and Modeling*.

[12] Cheng AC, Coleman RG, Smyth KT (2007). Structure-based maximal affinity model predicts small-molecule druggability. *Nature Biotechnology*.

[13] Bleakley K, Yamanishi Y (2009). Supervised prediction of drug-target interactions using bipartite local models. *Bioinformatics*.

[14] Zhao S, Li S (2010). Network-based relating pharmacological and genomic spaces for drug target identification. *PLoS One*.

[15] Wishart DS, Knox C, Guo AC (2008). DrugBank: a knowledgebase for drugs, drug actions and drug targets. *Nucleic Acids Research*.

[16] E. Stein S, R. Heller S, Tchekhovskoi D An open standard for chemical structure representation: the IUPAC chemical identifier.

[17] Bender A, Mussa HY, Glen RC, Reiling S (2004). Molecular similarity searching using atom environments, information-based feature selection, and a Naïve Bayesian classifier. *Journal of Chemical Information and Computer Sciences*.

[18] Keshava Prasad TS, Goel R, Kandasamy K (2009). Human protein reference database—2009 update. *Nucleic Acids Research*.

[19] Stark C, Breitkreutz BJ, Reguly T, Boucher L, Breitkreutz A, Tyers M (2006). BioGRID: a general repository for interaction datasets. *Nucleic Acids Research*.

[20] Aranda B, Achuthan P, Alam-Faruque Y (2009). The IntAct molecular interaction database in 2010. *Nucleic Acids Research*.

[21] Ceol A, Chatr Aryamontri A, Licata L (2009). MINT, the molecular interaction database: 2009 update. *Nucleic Acids Research*.

[22] Salwinski L, Miller CS, Smith AJ, Pettit FK, Bowie JU, Eisenberg D (2004). The database of interacting proteins: 2004 update. *Nucleic Acids Research*.

[23] Gibson TA, Goldberg DS (2009). Questioning the ubiquity of neofunctionalization. *PLoS Computational Biology*.

[24] Pinto YM, De Smet BGJL, Van Gilst WH (1993). Selective and time related activation of the cardiac renin-angiotensin system after experimental heart failure: relation to ventricular function and morphology. *Cardiovascular Research*.

[25] Kanehisa M, Goto S (2000). KEGG: Kyoto Encyclopedia of genes and genomes. *Nucleic Acids Research*.

[26] Zhao MW, Wang ED (2003). Regulation of angiogenic signaling pathway by two human aminoacyl-tRNA synthetases. *Progress in Biochemistry and Biophysics*.

[27] Cain AE, Tanner DM, Khalil RA (2002). Endothelin-1-induced enhancement of coronary smooth muscle contraction via MAPK-dependent and MAPK-independent [Ca^2+^]_i_ sensitization pathways. *Hypertension*.

[28] Schwartz RG, Barrett EJ, Francis CK (1985). Regulation of myocardial amino acid balance in the conscious dog. *Journal of Clinical Investigation*.

[29] Neutel JM (2010). Choosing among renin-angiotensin system blockers for the management of hypertension: from pharmacology to clinical efficacy. *Current medical research and opinion*.

[30] Ashburner M, Ball CA, Blake JA (2000). Gene ontology: tool for the unification of biology. *Nature Genetics*.

[31] Hase T, Tanaka H, Suzuki Y, Nakagawa S, Kitano H (2009). Structure of protein interaction networks and their implications on drug design. *PLoS Computational Biology*.

[32] Willenheimer R, Dahlöf B, Rydberg E, Erhardt L (1999). AT1-receptor blockers in hypertension and heart failure: clinical experience and future directions. *European Heart Journal*.

[33] Basile J, Toth PP (2009). Angiotensin receptor blockers: role in hypertension management, cardiovascular risk reduction, and nephropathy. *Southern Medical Journal*.

[34] Berl T (2009). Renal protection by inhibition of the renin-angiotensin-aldosterone system. *Journal of the Renin-Angiotensin-Aldosterone System*.

[35] Barbe F, Su JB, Guyene TT, Crozatier B, Ménard J, Hittinger L (1996). Bradykinin pathway is involved in acute hemodynamic effects of enalaprilat in dogs with heart failure. *American Journal of Physiology*.

[36] Rousseau MF, Konstam MA, Benedict CR (1994). Progression of left ventricular dysfunction secondary to coronary artery disease, sustained neurohormonal activation and effects of ibopamine therapy during long-term therapy with angiotensin-converting enzyme inhibitor. *American Journal of Cardiology*.

[37] Mehta PK, Griendling KK (2007). Angiotensin II cell signaling: physiological and pathological effects in the cardiovascular system. *American Journal of Physiology. Cell Physiology*.

[38] Beierwaltes WH (2010). The role of calcium in the regulation of renin secretion. *American Journal of Physiology. Renal Physiology*.

[39] Tang WHW, Parameswaran AC, Maroo AP, Francis GS (2005). Aldosterone receptor antagonists in the medical management of chronic heart failure. *Mayo Clinic Proceedings*.

